# Does Vitamin D Deficiency Lead to Hypertension?

**DOI:** 10.7759/cureus.1038

**Published:** 2017-02-17

**Authors:** Varshil Mehta, Shivika Agarwal

**Affiliations:** 1 Department of Cardiology, Mount Sinai Hospital, New York, USA; 2 Department of Forensic Medicine, ESIC Medical College, Faridabad, India

**Keywords:** risk factor, htn, vitamin supplementation, 25-hydroxy vitamin d

## Abstract

Hypertension (HTN) or high blood pressure is one of the most chronic and deadliest disorders in the world. There are many risk factors responsible for HTN which include age, race, using tobacco, high salt intake, etc. One of the risk factors we would like to highlight is low vitamin D levels. While there is strong evidence that Vitamin D plays an important role in maintaining bone and muscle health, there has been recent debate regarding its role in hypertension. However, there are many studies that have shown an indirect relation between 25-hydroxyvitamin D serum level and blood pressure. However, we suggest that more studies, especially randomised trials, should be conducted.

## Introduction and background

Hypertension or increase in blood pressure has been considered as one of the leading factors in causing worldwide disability-adjusted life years [1]. HTN has many etiological factors, which include age, race, family history, obesity, sedentary lifestyle, using tobacco, high salt intake, stress, and consuming alcohol in a larger quantity [2]. 

One of the recent studies by Kearney, et al. showed that more than one billion adults worldwide (approximately 25%) are suffering from HTN, and there is a possibility that this number will climb up to 29% by 2025 [3]. The prevalence rate of HTN among Iranian people aged 30 to 55 years old and those over 55 years old is approximately 23% and 50%, respectively [4].

Hypertension is considered as the fundamental reason for mortality on the planet, the most well-known reason for going to a doctor, and the least complex treatable and recognisable risk factor for diseases like cerebrovascular accident (CVA), myocardial infarction (MI), congestive heart failure (CHF), peripheral artery disease (PAD), atrial fibrillation (AF), and end-stage renal disease (ESRD) [5-6].

Regardless of solid archives showing the reality that treatment of hypertension will diminish the casualty significantly, hypertension is not treated or not totally treated in the vast majority of patients experiencing this issue in entire nations, including nations profiting from the most advanced therapeutic care frameworks. For this reason, hypertension is considered as one of the fundamental general medical problems [7].

In some observational reviews, it has been recognized that hypertension is more prevalent amid chilly months of a year as well as in zones that are a long way from the equator where sun radiation is reduced. For every 10 degrees of deviation from the equator, blood pressure and hypertension will rise 2.5 mm Hg and 2.5%, respectively [8-10].

More than 40% of African-Americans in the US experience hypertension while just 25% of white Americans have such an issue. African-Americans are prone to more severe and earlier hypertension, which further damages target organs and, subsequently, leads to premature disability and death [5, 11-12]. By this issue, we know that the decline in ultraviolet (UV) radiation, and henceforth, the decrease in the skin's capacity for Vitamin D synthesis, can have a connection with hypertension.

Krause, et al. utilized ultraviolet-B (UVB) light to treat patients with untreated mild essential hypertension (EH) and a deficiency of Vitamin D. These scientists found that UVB radiation, not UVA radiation, led to an increase in 25-hydroxyvitamin D (25(OH)D) levels and brought down blood pressure (BP) in Vitamin D-deficient patients with EH. Since 1998, this finding has brought extensive research enthusiasm up in the relationship between vitamin D inadequacy and EH [13].

Mehta suggested that low levels of Vitamin D, along with sugar and fats, should be considered as new risk factors in causing hypertension [14]. This article will bring forward the importance of Vitamin D and its role in maintaining blood pressure levels.

## Review

### Association of Vitamin D levels and hypertension

**Figure 1:**
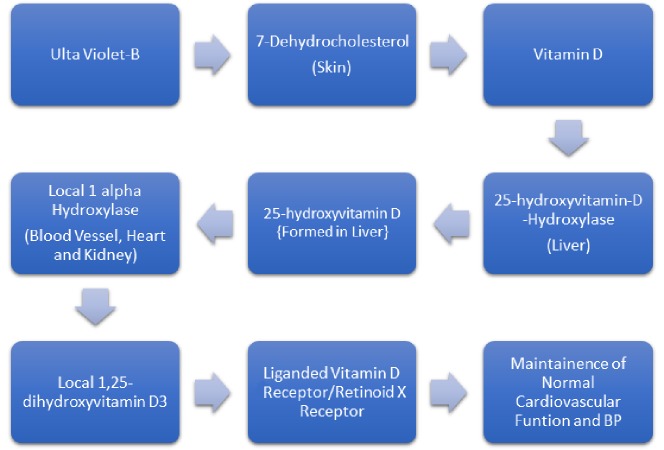
Role of Vitamin D in Maintaining Blood Pressure (15)

Ultraviolet-B from the sun prompts the development of pre-vitamin D3 from 7-hydrocholesterol in the skin, which then undergoes thermal isomerisation to form vitamin D3. It later undergoes hydroxylation to form 25-hydroxyvitamin D (25(OH)D) (by the action of 25-hydroxyvitamin-D-hydroxylase) in the liver and subsequently gets converted to 1,25-dihydroxyvitamin D3 (1,25 (OH_2_)D3) in the kidneys, blood vessels, and heart as shown in Figure 1 [15].

Many tissues and organs express VDR, including the heart, endothelium, vascular smooth muscle, and T cells [16-19].

VDR is a nuclear receptor to which Vitamin D3 (VD3) binds with high affinity and specificity. When bound with VD3, VDR is phosphorylated, which leads to a surface conformational change. This VDR is now activated and interacts with the retinoid X receptor (RXR) eventually forming a heterodimer that binds to Vitamin D-responsive elements in the region of gene promoter [20-21].

By recruiting either corepressors or coactivator complexes [22-23], activated VDR/RXR regulates the gene transcription for protein synthesis that modulates the traditional and non-traditional functions of VD3 (e.g., musculoskeletal health, calcium homeostasis, and normal BP and cardiovascular function) [24-25].

Along these lines, we can say that the role of Vitamin D in a cell, tissue, or organ relies on the local production or an adequate measure of VD3, expression levels of VDR/RXR and co-receptor proteins, and cell-specific transcriptional elements to manage genes that encode proteins altering Vitamin D signaling.

### Previous clinical studies

Kota, et al. demonstrated that systolic blood pressure, diastolic blood pressure, and mean arterial pressure had increased among individuals experiencing inadequacy of Vitamin D and proposed that Vitamin D deficiency is associated with renin-angiotensin-aldosterone system (RAAS) regulation [26]. One comparable review demonstrated that individuals with larger amounts of Vitamin D had lower blood pressure and a lower danger of developing hypertension [27].

A study done in 2013 took into account many reviews that included individuals with hypertension. They found that for every 10 ng/ml increment in Vitamin D levels, a 12% lower danger of developing hypertension was observed. The general population with the most noteworthy Vitamin D levels had a 30% lower danger of developing hypertension contrasted with the general population with the least levels. In any case, the greater part of the reviews was done in the United States, implying that we cannot know without a doubt if the outcomes would be the same in different countries [28].

A few studies have taken into account of the impact of Vitamin D on blood pressure in individuals who have hypertension. One such meta-analysis found out that taking Vitamin D supplementation could bring down systolic blood pressure [29].

A study by Jorde, et al. in 2010 exhibited a connection between low serum Vitamin D levels and hypertension. However, no confirmation was discovered showing that a Vitamin D prescription would have the capacity to prevent hypertension in the future [30].

A group of scientists studied the effect of taking a Vitamin D supplement on the risk of hypertension in African-Americans who had normal blood pressure was studied in 2013. These scientists appointed 250 individuals to get either 1,000 IU every day, 2,000 IU every day, 4,000 IU every day, or a placebo for three months. They discovered that for every increase in Vitamin D supplementation and Vitamin D levels in the body, systolic blood pressure declined; however, the diastolic pressure remained the same [31].

An experiment in 2012 in Denmark looked at the effects of Vitamin D supplements on lowering blood pressure in people with hypertension. For 20 weeks, people either took 3,000 IU per day of Vitamin D or a placebo pill. The researchers measured a few different types of blood pressure and found that the people in the Vitamin D group lowered their blood pressure more than the people getting the placebo. People in the Vitamin D group who had low levels of Vitamin D at the beginning of the study had a bigger reduction in their blood pressures. Vitamin D may be more effective in lowering blood pressure in people who have low levels of Vitamin D [32].

An Italian study conducted by Carrara, et al. in 2013 reviewed the role of Vitamin D supplementation on regulating the BP in the body. A group of hypertensives was given 25,000 IU per week of Vitamin D for a total of eight weeks. They found out that Vitamin D levels increased throughout the study and BPs were greatly reduced. Hence, they concluded that an overactive blood pressure system can lead to higher blood pressure and Vitamin D may help to reduce the risk of hypertension [33].

To test whether 25(OH)D levels are significantly associated with blood pressure and HTN risk, Vimaleswaran, et al. conducted a study (Mendelian randomization) and used different variants of genes that affect 25(OH)D synthesis or substrate availability (Vitamin D 25-hydroxylase and 7-dehydrocholesterol reductase) to meta-analyse 146,581 participants. They found out that each 10% increment in genetically instrumented 25(OH)D concentration was associated with a decrease in systolic BP (-0.37 mmHg, P = 0.052) and diastolic BP (-0.29 mmHg, P = 0.01), and an 8.1% reduced odds of HTN (P = 0.002). The findings of a study conducted later, further confirmed that increased 25(OH)D concentrations might decrease the risk of HTN [34-35].

Caro, et al. concluded that serum 25(OH) Vitamin D levels do not have any significant statistical association with blood pressure [36]. In 2008, an experiment used data from a large experiment that assigned women to receive either 1-gram per day of calcium, plus 400 IU per day of Vitamin D, or a placebo pill. They observed that there was no significant difference in blood pressure changes between both the groups [37].

It was shown in a review done by Lee, et al. that serum Vitamin D and parathyroid hormone (PTH) levels have no significant connection in relation to hypertension among Chinese individuals [38]. In a cross-sectional review of 251 individuals (age 40 or more years old) by Kashi, et al., it was discovered that there was no association between hypertension and serum 25(OH) Vitamin D, calcium, and PTH levels [39].

Some other unknown elements might have an impact on the relation between Vitamin D and hypertension, especially in older age groups, as Sanijder reported in his review, which showed Vitamin D effect’s on blood pressure might be indirectly based on its role in parathyroid hormone performance [40]. The comparison and findings of all the studies are presented in Table 1.

**Table 1:** Comparison and Findings of the Studies 25(OH)D: 25-hydroxyvitamin D; HTN: hypertension

Author	Year of Publication	N	Mean Age (Years)	Mean 25(OH)D (ng/ml)	Mean Systolic Blood Pressure (mm/Hg)	Mean Diastolic Blood Pressure (mm/Hg)	Findings
Kota, et al. [26]	2011	50	49.5 ± 7.8	18.5 ± 6.4	162.4 ± 20.2	100.2 ± 11.2	Lower 25(OH)D levels associated with increased blood pressure
Ullah, et al. [27]	2010	-	-	-	-	-	Larger 25(OH)D levels associated with lower blood pressure
Kunutsor, et al. [28]	2013	-	-	-	-	-	Larger 25(OH)D levels associated with lower chances to develop HTN
Witham, et al. [29]	2009	-	-	-	-	-	Larger 25(OH)D levels associated with lower chances to develop HTN
Jorde, et al. [30]	2010	4,125	-	-	-	-	No association between 25(OH)D and HTN
Forman, et al. [31]	2007	117,730	-	-	-	-	Larger 25(OH)D levels associated with lower chances to develop HTN
Larsen, et al. [32]	2012	112	61 ± 10	23 ± 10	Decreased by 7 mmHg after taking Vitamin D supplementation in 24 hrs vs. placebo	Decreased by 2 mmHg after taking Vitamin D supplementation vs. placebo	Vitamin D supplementation in patients with low levels of 25(OH)D brings down blood pressure
Carrara, et al. [33]	2013	15	43.6 ± 21.6	18.3 ± 2.8	At 0 week: 137.4 ± 1.8	At 0 week: 81.6 ± 1.8	Vitamin D supplementation in patients with low levels of 25(OH)D brings down blood pressure
					At 8 weeks (after Vitamin D supplementation): 134.8 ± 2.3	At 8 weeks: 81.0 ± 1.6	
Vimaleswaran, et al. [34]	2014	146,581	-	-	-	-	Larger 25(OH)D levels associated with lower blood pressure
Caro, et al. [36]	2012	219	41.5 ± 13.9	29.2 ± 10.6	113.2 ± 13.1	73.3 ± 9.7	No association between 25(OH)D and HTN
Margolis, et al. [37]	2008	36,282	62 ± 7	-	-	-	No association between 25(OH)D and HTN
Li, et al. [38]	2012	1,420	-	-	-	-	No association between 25(OH)D and HTN
Kashi, et al. [39]	2004	251	50 ± 8	-	-	-	No association between 25(OH)D and HTN

## Conclusions

Hypertension is one of the most crucial chronic diseases, which can cause many other cardiovascular disorders and eventually may lead to death. This article reviews whether serum Vitamin D concentration plays an important role in causing hypertension or not. Because of the opposing consequences of different reviews on the role of Vitamin D in preventing hypertension development or its treatment, it appears that Vitamin D levels in the body modulate the blood pressure indirectly. More studies should be conducted after eliminating the compounding factors in order to prove the association. We suggest that physicians should keep a check on the Vitamin D levels of their patients in order to curb the ever-increasing incidence of hypertension.
